# Burnout Among Italian Medical Doctors: A Cross-Sectional Study on Emotional Exhaustion, Depersonalization, and Gratification Post-COVID-19

**DOI:** 10.3390/healthcare14040454

**Published:** 2026-02-11

**Authors:** Francesco Leonforte, Marco Sapienza, Martina Ilardo, Klara Komici, Cristina Madaudo, Claudio Sanfilippo, Vito Nicosia, Fabio Raciti, Antonio Mistretta, Vito Pavone, Gianluca Testa

**Affiliations:** 1 Department of Integrated Hygiene, Organizational, and Service Activities (Structural Department), Health Management, University Hospital Polyclinic “G. Rodolico—San Marco”, 95123 Catania, Italy; leonfortefrancesco1@gmail.com; 2 Department of General Surgery and Medical Surgical Specialties, University Hospital Polyclinic “G. Rodolico—San Marco”, 95123 Catania, Italy; martinailardo52@gmail.com (M.I.); fabio_raciti@icloud.com (F.R.); vitopavone@hotmail.com (V.P.); 3 Department of Medicine and Health Sciences, University of Molise, 86100 Campobasso, Italy; klara.komici@unimol.it; 4 Department of Health Promotion, Mother and Child Care, Internal Medicine and Medical Specialties (ProMISE), University of Palermo, 90127 Palermo, Italy; cristina.madaudo@unipa.it; 5 Division of Cardiology, Centro Cuore Morgagni, 95030 Catania, Italy; claudiosanfilippo13@gmail.com; 6 Department of Medical and Surgical Sciences and Advanced Technologies “G.F. Ingrassia”, University of Catania, 95123 Catania, Italy; vitonicosia6@gmail.com (V.N.); anmist@unict.it (A.M.)

**Keywords:** burnout, emotional exhaustion, healthcare workers, COVID-19 pandemic, Maslach Burnout Inventory, GHQ-12, occupational health

## Abstract

**Background:** Burnout is a growing concern among medical doctors, particularly in high-pressure environments, which has been exacerbated by the COVID-19 pandemic. This study investigates the prevalence and determinants of burnout among physicians working in a large hospital in Southern Italy. **Methods:** This online cross-sectional survey evaluated burnout and emotional distress among physicians and trainees at Catania Hospital using the Maslach Burnout Inventory (MBI) and General Health Questionnaire (GHQ-12). Data collection (July–August 2025) incorporated strict anonymity to mitigate social desirability bias. Statistical analyses, including chi-squared tests with Tukey adjustments and Pearson correlations, were stratified by sex and specialization area to identify significant psychological associations. **Results:** High levels of burnout were observed across all dimensions: 76.7% of respondents reported low personal gratification, 70.8% showed high depersonalization, and nearly 50% experienced high emotional exhaustion. Female physicians and recent graduates (after 2020) exhibited significantly lower levels of gratification and higher psychological distress. Service-area professionals reported lower emotional exhaustion, but also lower gratification compared to surgical specialties. Notably, otorhinolaryngology showed both the highest burnout risk and the highest gratification scores. **Conclusions:** Burnout is alarmingly prevalent among Italian medical doctors, and there is significant variation across sexes, graduation cohorts, and medical specialties. Despite the high burnout levels identified, the cross-sectional design and non-probability sampling necessitate a cautious interpretation of these findings. Future longitudinal research involving larger, more representative cohorts is essential to validate these results and inform targeted institutional interventions.

## 1. Introduction

Burnout is a psychological syndrome that results from chronic workplace stress that has not been successfully managed. It is characterized by three dimensions: emotional exhaustion, depersonalization, and a reduced sense of gratification. This condition is prevalent in professions that require constant interaction with people, such as healthcare and education, and can lead to various emotional, behavioral, and psychosomatic issues [1,2]. Burnout negatively impacts both individual performance and overall well-being, and in the workplace, it can lead to decreased job satisfaction and performance [3,4]. Studies indicate that approximately 40% of mental health professionals experience high levels of emotional exhaustion, while depersonalization and low gratification are also significant concerns [5,6].

Burnout is prevalent among healthcare workers globally, and estimates suggest that more than half of physicians experience burnout symptoms, which adversely affect patient care and healthcare systems [7,8,9]. Several factors contribute to burnout among healthcare workers. Significant organizational drivers include excessive workloads, inefficient work processes, and work-home conflicts [8,9]. Individual factors such as younger age, less work experience, and being unmarried are also associated with higher burnout levels [10]. Additionally, workplace factors like workload, job autonomy, and perceived support from leadership play important roles in predicting burnout [11].

Burnout has severe implications for healthcare systems, including reduced job performance, lower career satisfaction, and increased vulnerability among healthcare workers [9,12]. It also poses risks to public health by affecting the effectiveness and availability of healthcare providers, which ultimately compromises patient safety and care quality [8,9]. Burnout negatively impacts both physical and mental health and leads to symptoms such as depression, anxiety, sleep disturbances, and memory impairment [13]. It also affects job performance, interpersonal relationships, and increases vulnerability to illnesses [14,15].

During the COVID-19 pandemic, burnout rates among those who worked in the intensive care unit and emergency department ranged from 49.3% to 58% [16]. The pandemic has significantly increased the workload and stress levels of healthcare workers, leading to a heightened prevalence of burnout. The prevalence of burnout among healthcare workers during the pandemic has been notably high, and studies indicate that nearly half of them experienced burnout. Emotional exhaustion, depersonalization, and a lack of gratification were common, among which emotional exhaustion and depersonalization have affected over 50% of workers according to studies [17,18,19]. Factors such as long shifts, redeployment, and pre-existing psychological issues also contribute to increased burnout levels [18,19].

In Italy, burnout is notably prevalent among healthcare workers as well, and high rates have been observed in emergency departments. Physicians in these settings report burnout rates ranging from 45 to 55%, and emotional exhaustion and depersonalization are common symptoms [20]. Factors contributing to burnout include high job demands, lack of resources, and challenging work environments [20,21].

The work environment plays a critical role in the development of burnout. In Southern Italy, healthcare workers have reported poor environmental conditions, such as inadequate equipment handling and lack of privacy, which are associated with higher burnout levels, particularly emotional exhaustion and depersonalization [21]. Sex differences in burnout have also been observed, with female healthcare workers experiencing higher levels of emotional exhaustion [22]. Italian healthcare workers have experienced high levels of burnout, including significant emotional exhaustion, depersonalization, and reduced gratification, especially among those in emergency departments and intensive care units [19,20,23]. Therefore, this study investigates the prevalence and determinants of burnout among physicians working in a large hospital in Southern Italy.

## 2. Materials and Methods

This cross-sectional survey study examined physicians and trainees in all specialties working in Catania Hospital. Family doctors, trainees in family medicine, students, and nursing staff were excluded from this study. Based on the results of a previous survey, the sample size was set as 82 participants considering power = 0.80 and α = 0.05 [24].

A survey was developed based on Burns’ methodology [25] and included demographic information such as age, sex, marital status, number of children, year of graduation, specialization area, working hours per week, and working department. Furthermore, we applied the Italian version of Maslach Burnout Inventory (MBI), (Mind Garden Inc., Menlo Park, CA, USA), a questionnaire validated among healthcare professionals evaluating three key indicators: emotional exhaustion, personal gratification, and depersonalization. Regarding emotional exhaustion, a total score of 17 or less indicates low burnout level, while 18–29 indicates moderate burnout, and 30 or above indicates a high burnout level. A personal gratification score of 33 or less indicates high-level burnout, 34–39 indicates moderate burnout, and 40 or above indicates low burnout. A depersonalization score of 5 or indicates less low-level burnout, 6–11 indicates moderate burnout, and 12 or above indicates a high burnout level [26,27].

In addition, the Italian version of General Health Questionnaire (GHQ-12), a validated instrument designed to detect minor psychiatric disorders or emotional distress in the general population, was also applied in the survey. The questionnaire comprises 12 items that assess a variety of psychological symptoms, including sleep, tension, temperament, and social functioning difficulties [28,29]. The questionnaire was distributed via an online survey tool (Google Forms; Google LLC, Mountain View, CA, USA) from July to August 2025, and all data were collected anonymously. This study was approved by the Institutional Review Board of the University of Catania (46/2025/PAR). Participants received the study information and implied consent forms before starting the survey. To mitigate the risk of social desirability bias, participants were explicitly guaranteed full anonymity at every stage of the study, and instructions emphasized that there were no right or wrong answers, thereby encouraging honest reporting and reducing evaluative apprehension.

### Statistical Analysis

Descriptive statistics were applied, and data were expressed as numbers, percentages, means, and standard deviations (SDs). The chi-squared test with Tukey adjustment for multiple comparisons was performed. The analysis was stratified according to sex and specialization areas. Pearson’s correlation matrix was assessed to verify the correlation between different key indicators of burnout. Eventual missing data were handled using listwise deletion, where cases were excluded from all analyses if there were missing data. Data were analyzed using STATA software (StataCorp LLC, College Station, TX, USA), and statistical significance was set at *p* < 0.05.

## 3. Results

### 3.1. Population Characteristics

**Figure 1:**
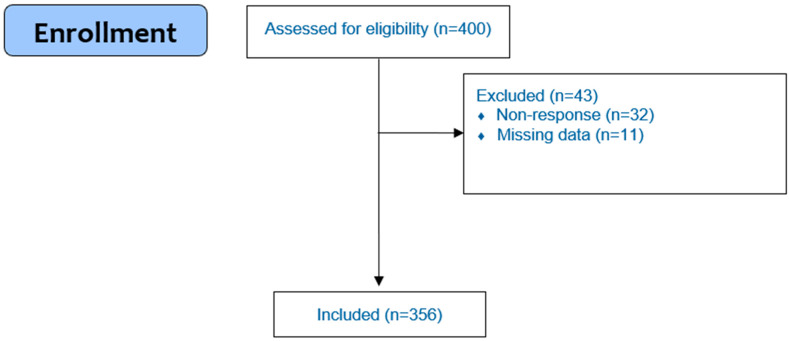
Study participation flow diagram.

A cohort of 400 physicians and trainees across all specialties at Catania Hospital was recruited via Department Heads. A total of 367 participants completed the survey, yielding a 91.8% response rate. This high engagement is attributed to the clinical relevance of the study topic; conversely, non-responses were primarily due to the inherent limitations of online self-administration. Ultimately, 356 participants were deemed eligible after deletion of surveys with missing data (Figure 1). The mean age of participants was 34.16 ± 9.2 years, 51.2% were male, and 48.8% were female. Regarding marital status, 41.7% were married or cohabitating, 57.2% were not married or cohabitating, and only 1.1% were divorced or single. Furthermore, 80.9% were childless, and only 20.1% had one or more children. Most of the respondents were resident physicians (65.1%). A total of 74.6% of respondents worked more than 40 h per week, and only 26.4% worked 40 h per week or less. The characteristics of the participants are reported in Table 1.

### 3.2. Burnout Level and Correlation Between Domains

Overall, the survey results are rather concerning as all four indicators of well-being show high risk levels for at least 50% of the sample. The dimension showing the most critical results was gratification: only 4.8% of respondents felt gratified in their work, while more than three-quarters (76.7%) reported low levels of gratification. Another concerning domain was depersonalization: just 7.3% of participants reported little or no signs of depersonalization, and 70.8% fell into the high-risk category for this indicator.

**Figure 2:**
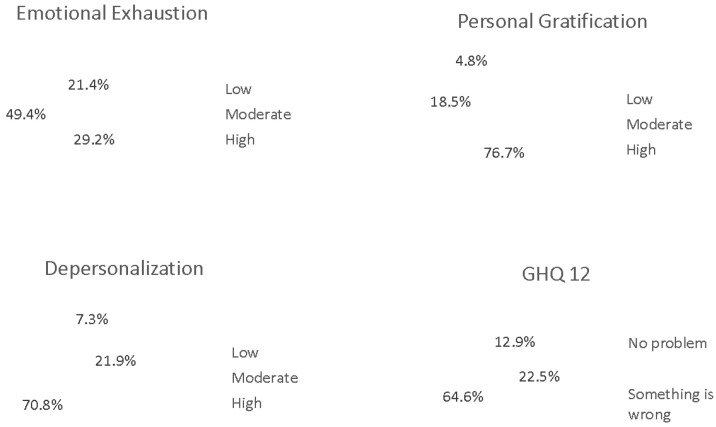
Burnout level in overall population (“low personal accomplishment/gratification = higher burnout risk”).

The third dimension of the MBI, emotional exhaustion, was rated as high by nearly 50% of the sample, while only 21% reported experiencing mild emotional fatigue. Consistent with these findings, the results of the GHQ-12 further reflect a general trend of distress within the sample: 64.6% of respondents showed signs of needing support, and only 12.9% reported experiencing no psychological distress (Figure 2). The Pearson analysis revealed a significant positive correlation between the perception of general health and emotional exhaustion, depersonalization, and gratification (Table 2). The observed dissociation between personal gratification and the other subscales confirms that low personal accomplishment is not merely a consequence of exhaustion or depersonalization, but rather a distinct psychological process linked to professional efficacy (Table 2).

### 3.3. Graduation Year, Sex, and Specialization

Stratification according to graduation year was performed considering that since 2020, the single-cycle master’s degree in medicine and surgery became a direct qualification for the practice of medicine in Italy. Graduates are no longer required to pass a national licensing exam to obtain professional certification, provided they have received a positive evaluation in the practical internship during their studies (covering medical, surgical, and general practice areas). This change has facilitated immediate entry into the medical workforce, which was under immense pressure at the beginning of 2020 due to the outbreak of the COVID-19 pandemic [30]. Our data indicate that emotional exhaustion, depersonalization, and perception of general health were not statistically different among physicians who graduated after 2020 compared to those who graduated before (*p* = 0.19, *p* = 0.97, and *p* = 0.97). The gratification level was higher among physicians who graduated before 2020 (*p* < 0.01), as shown in Table 3, with less risk of burnout. After stratifying by sex (Table 4, there were no significant differences regarding emotional exhaustion (*p* = 0.09) and depersonalization (*p* = 0.97), that are link with a higher risk of burnout. However, females had lower levels of gratification compared to males (*p* < 0.01), and the perception of health according to the GHQ-12 was worse among females (*p* = 0.05). Regarding areas of professional practice, those working in service areas reported lower levels of emotional exhaustion compared to those in surgery (*p* = 0.025), where increase the possibility of burnout.

Despite not being statistically significant, the level of emotional exhaustion was also lower compared to those working clinical areas (*p* = 0.079), as shown in Table 5. Despite gratification being more frequent among professionals in surgery (*p* = 0.04), their level of depersonalization was higher (*p* = 0.01). No substantial differences emerged between the surgical and clinical medicine areas. Regarding general well-being, no statistically significant differences were observed among any of the professional areas examined.

### 3.4. Burnout Level and General Health Across Different Medical Specialties

Due to the limited sample size, it was not possible to achieve comprehensive representativeness for each specialty. Therefore, only specialties with at least five respondents in the sample were included in the descriptive analysis. In terms of emotional exhaustion, the specialties with the highest reported risks were otorhinolaryngology (with an average score of 1.75), general surgery (1.6), and pediatrics (1.58). On the other hand, the lowest risks were found in geriatrics (0.6) and hygiene and preventive medicine (0.74). Regarding depersonalization, the specialties with the highest reported risks were otorhinolaryngology (with an average of 2), pediatrics (1.89), and general surgery (1.85). The lowest risks were seen in ophthalmology (1.33), occupational medicine (1), and hygiene and preventive medicine (0.78).

In terms of personal gratification, the specialties with the lowest scores were urology (0), anesthesia and intensive care (0.06), and pneumology (0.11). In contrast, the specialties with the highest levels of gratification were otorhinolaryngology (0.62), psychiatry (0.53), and occupational medicine (0.5). Finally, when evaluating general well-being, the specialties with the lowest levels were pediatrics (with an average of 1.84), followed by pneumology (1.78) and a tie between neurology and otorhinolaryngology (1.75). The specialties with the highest general well-being scores were geriatrics (1.2), dermatology (1.17), and occupational medicine (0.67). Interestingly, the otorhinolaryngology specialty showed the highest risks in terms of general well-being, but it also had the highest levels of personal gratification. These data are presented in Table 1, Table 2, Table 3 and Table 4.

## 4. Discussion

We assessed burnout levels using three key indicators derived from responses to the MBI questionnaire: emotional exhaustion, personal gratification, and depersonalization. Additionally, a fourth indicator, general well-being, was evaluated based on responses to the GHQ-12 [27]. The level of burnout was evaluated using a three dimensions-scoring system. The results were interpreted as shown in Table 6.

The Maslach Burnout Inventory (MBI) is a questionnaire consisting of 22 items, each with 6 response levels on a Likert scale [27]; a higher score indicates higher levels of burnout for Emotional Exhaustion and Depersonalization, while for gratification, a lower score indicates higher burnout. Among the four indicators considered, the only statistically significant difference in terms of graduation year was found for the dimension of personal gratification. Specifically, individuals who graduated in 2020 or later reported significantly lower levels of personal gratification compared to those who completed their degree prior to 2020. The transition from medical student to junior doctor is a challenging period marked by increased clinical responsibilities, steep learning curves, and psychological stressors. Many young doctors face poor working conditions and a lack of organizational support, which can impact their sense of purpose and job satisfaction [31]. Therefore, a significant majority of new medical graduates feel unprepared to work independently immediately after graduation. Approximately 97% of graduates express a need for mentorship, and 80.5% prefer to work with a mentor for a year or more. This highlights the importance of structured support systems to help young doctors transition smoothly into their professional roles [32]. Additionally, the choice of medical specialty among young doctors is influenced by several factors, including remuneration, work–life balance, and personal motivations. Flexibility in working hours and better working conditions are also important considerations. Challenges such as limited residency slots and a lack of knowledge about specific specialties can hinder the decision-making process [33]. No striking differences were observed for sex. However, a statistically significant difference was detected at the 10% confidence level for emotional exhaustion and general well-being (GHQ-12). In both cases, women reported higher levels of risk than men. Research consistently indicates that female physicians experience higher levels of emotional exhaustion than their male counterparts. This is a significant dimension of burnout that is prevalent among physicians regardless of their specialty or work environment [34,35,36].

Emotional exhaustion is linked to a higher risk of depression and anxiety, which can impact overall mental health and job satisfaction [37]. A study conducted in a tertiary care hospital in Pakistan found that female doctors reported feeling more emotionally drained and fatigued than male doctors, particularly during the COVID-19 pandemic [38]. Similarly, a systematic review highlighted that female physicians are more likely to experience burnout than male physicians, especially in the emotional exhaustion dimension [32]. This trend is also observed in emergency physicians, among whom females reported higher emotional exhaustion and lower personal accomplishment than males [39]. This aligns with findings from other studies that suggest women in healthcare roles, including physicians, have a higher risk of experiencing poor mental health outcomes due to increased emotional exhaustion [40,41].

Professionals working in the service area reported a lower risk of emotional exhaustion and depersonalization than those working in clinical medicine and surgical specialties. However, they also showed lower levels of personal gratification, particularly when compared to those in the surgical field. The data suggest that burnout varies significantly across medical specialties, especially in terms of emotional exhaustion and depersonalization. The service area, which may include non-surgical and non-clinical roles, tended to report lower levels of emotional exhaustion and depersonalization. This could be due to different work demands and stressors compared to clinical and surgical environments [42,43]. Compared to other medical fields, surgeons and surgical residents often report higher levels of burnout, particularly in terms of emotional exhaustion and depersonalization [44,45,46]. Similarly, emergency medicine physicians and medical residents also experience significant levels of emotional exhaustion and depersonalization, which contribute to poor quality of care and increased medical errors [44,47,48]. The intensity of clinical practice and the risk of litigation further exacerbate these conditions [48]. However, no substantial differences emerged between the surgical and clinical medicine areas. Additionally, when considering the variable of general well-being, no statistically significant differences were observed among any of the professional areas examined.

Interestingly, the otorhinolaryngology specialty showed the highest risks in terms of general well-being but also had the highest levels of personal gratification. This indicates that the intensity and demands of the work are associated with high personal satisfaction. Despite the high risk of burnout, otorhinolaryngology offers significant professional gratification. Satisfaction with surgical skills and specialty choice is positively associated with higher levels of emotional intelligence and a sense of personal gratification [49].

## 5. Conclusions

This study had methodological and interpretative limitations that must be acknowledged. Firstly, the sample may not be fully representative of the broader population of healthcare workers, since the selection was not carried out using probabilistic methods. As a result, certain professional categories, age groups, sexes, or geographic and organizational contexts (such as public vs. private sectors or hospital vs. community care) may be over or under-represented. There is also a potential selection bias: individuals experiencing higher levels of stress or burnout may have been more motivated to participate, while those with more severe symptoms may not have had the time or energy to complete the survey. This dual effect can distort the true prevalence of burnout by either inflating or under-representing its extent and for this reason, these results require a cautious interpretation.

Secondly, the relatively limited sample size also reduces the statistical power of the study, particularly in the subgroup analyses. Small numbers in specific specialties or demographic groups may result in increased variability and limit the reliability and robustness of the findings. Additionally, the use of self-reported questionnaires introduces inherent subjectivity, even though they were based on validated instruments such as the MBI and GHQ-12. Respondents’ answers may have been influenced by temporary emotional states, potentially leading to an underreporting of symptoms.

Another important limitation concerns the cross-sectional design of the study, which provides a snapshot at a single point in time. Finally, the generalizability of the findings is limited. For these reasons, the results should be interpreted with caution, and further studies with larger, more representative samples and longitudinal designs are needed to better understand the dynamics of burnout among In conclusion, this study has highlighted a concerning level of burnout among medical doctors, and significant proportions of the sample experienced high emotional exhaustion, depersonalization, and low personal gratification. The most alarming finding relates to gratification: over three-quarters of respondents reported low levels of satisfaction with their work. Emotional exhaustion affected nearly half of participants, and more than two-thirds reported high levels of depersonalization.

Recent medical graduates—particularly those who completed their degrees after 2020—showed significantly lower levels of personal gratification. This likely reflects the abrupt and high-pressure transition into the medical workforce during the COVID-19 pandemic and underscores the need for structured support and mentorship during the early stages of medical careers. Sex differences, while not dramatic, revealed that women are at greater risk for emotional exhaustion and psychological distress, echoing other research on sex-based disparities in healthcare burnout. Differences also emerged across professional areas: workers in service-oriented roles reported lower emotional strain but also experienced lower job satisfaction, especially when compared to surgical specialties. Burnout risks also varied considerably according to medical specialty, and otorhinolaryngology, general surgery, and pediatrics were the most affected in terms of emotional exhaustion and depersonalization. However, otorhinolaryngology stood out as the specialty with both the highest levels of burnout and the highest levels of personal gratification, suggesting that while the work is demanding, it may also offer strong professional rewards.

Overall, these results point to the urgent need for targeted measures to address burnout, such as improved working conditions, better work–life balance, accessible mental health support, and tailored strategies that account for specialty- and demographic-specific vulnerabilities. Further research with broader and more diverse samples is recommended to strengthen these findings and inform policy and organizational change.

## Figures and Tables

**Table 1:** Participant characteristics (total population *n* = 356).

	Participant Characteristics (n = 356)	
Sex n (%)	Male	184 (51.69)
	Female	172 (48.31)
Age mean, SD (range)		34.16 ± 9.2 (25–66)
Marital status n (%)	Single or not cohabiting	208 (58.43)
	Married or cohabiting	148 (41.57)
	0	289 (81.18)
Number of Children n (%)	1	27 (7.58)
	2	35 (9.83)
	≥3	5 (1.40)
	≤40	93 (26.12)
Working Hours n (%)	41–50	163 (45.79)
	51–60	100 (28.09)
	Services area	92 (25.92)
Medical Specialization Areas n (%)	Medical area	152 (42.82)
	Surgical area	111 (31.27)

SD: standard deviation; n: number.

**Table 2:** Correlation matrix.

Variables	GHQ 12	Emotional Exhaustion	Depersonalization	Gratification
GHQ 12	1.000			
Emotional Exhaustion	0.588 *	1.000		
	(0.000)			
Depersonalization	0.373 *	0.591 *	1.000	
	(0.000)	(0.000)		
Gratification	−0.207 *	−0.020	0.013	1.000
	(0.000)	(0.706)	(0.811)	

* *p* < 0.01.

**Table 3:** Analysis stratified by year of graduation.

		**Emotional Exhaustion**		
	Low	Moderate	High	*p*-value
Graduation before 2020	32 (42.11)	42 (40.38)	87 (49.43)	0.19
Graduation after 2020	44 (57.89)	62 (59.62)	89 (50.57)	
		**Depersonalization**		
	Low	Moderate	High	*p*-value
Graduation before 2020	12 (46.15)	35 (44.87)	114 (45.24)	0.97
Graduation after 2020	14 (7.18)	43 (55.13)	138 (54.76)	
		**Gratification**		
	Low	Moderate	High	*p*-value
Graduation before 2020	109 (39.93)	40 (60.61)	12 (70.59)	*p* < 0.01
Graduation after 2020	164 (60.07)	26 (39.39)	29 (41)	
		**GHQ 12**		
	No problem	Something is wrong	I need help	*p*-value
Graduation before 2020	23 (50)	32 (40)	106 (46.09)	*p* = 0.97
Graduation after 2020	23 (50)	48 (60)	124 (53.91)	

Data are expressed as number with percentage in brackets.

**Table 4:** Analysis stratified by sex.

		**Emotional Exhaustion**		
	Low	Moderate	High	*p*-value
Male (n = 184)	42 (55.26)	61 (58.65)	81 (46.02)	0.09
Female (n = 172)	34 (44.74)	43 (41.35)	95 (53.98)	
		**Depersonalization**		
	Low	Moderate	High	*p*-value
Male (n = 184)	12 (46.15)	35 (44.87)	114 (45.24)	0.97
Female (n = 172)	14 (7.18)	43 (55.13)	138 (54.76)	
		**Gratification**		
	Low	Moderate	High	*p*-value
Male (n = 184)	109 (39.93)	40 (60.61)	12 (70.59)	*p* < 0.01
Female (n = 172)	164 (60.07)	26 (39.39)	29 (41)	
		**GHQ 12**		
	No problem	Something is wrong	I need help	*p*-value
Male (n = 184)	29 (63.04)	44 (55)	111 (48.26)	*p* = 0.05
Female (n = 172)	17 (36.96)	36 (45)	119 (51.74)	

Data are expressed as numbers with percentages in brackets (higher percentages of emotional exhaustion, depersonalization, gratification, and GHQ12 indicate higher risk of burnout).

**Table 5:** Analysis stratified by areas of professional practice.

		**Emotional Exhaustion**		
	Low	Moderate	High	*p*-value
Medical (n = 152)	32 (42.67)	39 (37.50)	81 (46.02)	*p* = 0.025 (services vs. surgery)
Surgery (n = 111)	18 (24)	32 (30.77)	61 (34.66)	*p* = 0.78 (clinical vs. surgery)
Services (n = 92)	25 (33.3)	33 (31.73)	34 (19.32)	*p* = 0.079 (clinical vs. services)
		**Depersonalization**		
	Low	Moderate	High	*p*-value
Medical (n = 152)	6 (23.08)	26 (33.77)	120 (47.62)	*p* = 0.01 (services vs. surgery)
Surgery (n = 111)	6 (23.08)	22 (28.57)	83 (32.94)	*p* = 0.73 (clinical vs. surgery)
Services (n = 92)	14 (53.85)	29 (37.66)	49 (19.44)	*p* < 0.001 (clinical vs. services)
		**Gratification**		
	Low	Moderate	High	*p*-value
Medical (n = 152)	115 (42.28)	30 (45.45)	7 (41.18)	*p =* 0.04 (services vs. surgery)
Surgery (n = 111)	79 (29.04)	24 (36.36)	8 (47.06)	*p =* 0.54 (clinical vs. surgery)
Services (n = 92)	78 (28.68)	12 (18.18)	2 (11.76)	*p =* 0.24 (clinical vs. services)
		**GHQ 12**		
	No problem	Something is wrong	I need help	*p*-value
Medical (n = 152)	16 (35.56)	34 (42.50)	102 (44.35)	*p =* 0.59 (services vs. surgery)
Surgery (n = 111)	17 (36.96)	36 (45)	119 (51.74)	*p =* 0.92 (clinical vs. surgery)
Services (n = 92)	15 (33.33)	22 (27.50)	55 (23.91)	*p =* 0.34 (clinical vs. services)

Data are expressed as numbers with percentages in brackets (higher percentages of emotional exhaustion, depersonalization, gratification, and GHQ12 indicate higher risk of burnout).

**Table 6:** Interpretation of MBI results.

MBI	Low-Level Burnout	Moderate Burnout	High-Level Burnout
**Emotional Exhaustion**	Total score of 17 or less	Total score between 18 and 29 (inclusive)	Total score over 30
**Depersonalization**	Total score of 5 or less	Total score between 6 and 11 (inclusive)	Total score of 12 or greater
**Gratification**	Total score of 33 or less	Total score between 34 and 39 (inclusive)	Total score greater than 40

## Data Availability

The data presented in this study are not publicly available due to ethical and privacy restrictions. Data are available from the corresponding author upon reasonable request and with permission of the local Ethics Committee.
